# Study of Mid-Infrared Emission and Structural Properties of Heavy Metal Oxide Glass and Optical Fibre Co-Doped with Ho^3+^/Yb^3+^ Ions

**DOI:** 10.3390/ma12081238

**Published:** 2019-04-15

**Authors:** Tomasz Ragin, Agata Baranowska, Marcin Kochanowicz, Jacek Zmojda, Piotr Miluski, Dominik Dorosz

**Affiliations:** 1 Faculty of Mechanical Engineering, Bialystok University of Technology, Wiejska 45c, 15-351 Bialystok, Poland; a.baranowska@doktoranci.pb.edu.pl; 2 Faculty of Electrical Engineering, Bialystok University of Technology, Wiejska 45d, 15-351 Bialystok, Poland; m.kochanowicz@pb.edu.pl (M.K.); j.zmojda@pb.edu.pl (J.Z.); p.miluski@pb.edu.pl (P.M.); 3 Faculty of Materials Science and Ceramics, AGH University of Science and Technology, Mickiewicza 30, 30-059 Krakow, Poland; ddorosz@agh.edu.pl

**Keywords:** mid-infrared emission, bismuth-germanate glass, heavy metal oxide, 2.87 µm luminescence, Ho^3+^/Yb^3+^, optical active fibre

## Abstract

Bismuth-germanate glasses with low hydroxide content co-doped with Ho^3+^/Yb^3+^ ions have been investigated in terms of structural and spectroscopic properties. To reduce OH^-^ ions content and improve transmittance value at the wavelength of 3.1 µm, the glass synthesis has been carried out in low vacuum conditions (45–65 mBar). The composition of the host glass based on heavy metal oxides affects the maximum phonon energy (hω_max_ = 724 cm^−1^), which low value has a positive impact on the mid-infrared emission parameters. Emission band at the wavelength of 2.87 µm was observed in glass co-doped with mol% 0.25 Ho_2_O_3_/0.75 Yb_2_O_3_ under 980 nm high power laser diode wavelength excitation. Lifetime measurements of the Yb^3+^:^2^F_5/2_ quantum level indicate efficient Yb^3+^ → Ho^3+^ energy transfer (η = 61%). The developed active bismuth-germanate glass was used as the core of optical fibre operating in the mid-infrared region.

## 1. Introduction

Research on new optical materials for the mid-infrared region has been conducted for many years due to their potential use in applications such as atmosphere pollution monitoring, eye-safe laser radar, remote sensing, and microsurgery [1,2,3,4]. The spectral transmittance in the mid-IR region of most common optical glasses based on oxides such as phosphate (1200 cm^−1^), silicate (1100 cm^−1^), or borate (1400 cm^−1^) is limited due to their high maximum phonon energies [5,6]. On the other hand, non-oxide glasses with low phonon energy and high transparency in mid-IR are characterized by poor thermal stability which complicates processing them into photonic structures [7,8,9,10,11]. Thus, glasses based on heavy metal oxides (HMO) are still investigated in optical material laboratories around the world [12,13,14]. Especially, bismuth-oxide based glasses characterized by relatively low maximum phonon energy, wide transparency windows, high thermal stability, and good mechanical and chemical properties are considered for use in optical fibre technology [15,16,17,18]. However, in the wavelength range near 3 μm exists an absorption band, which originates from hydroxide ions. In order to minimalize its adverse impact on the transmittance properties, the glass synthesis process has to be conducted in low vacuum conditions [19].

Among rare earth ions, holmium ions are characterized by the luminescence band at the wavelength of 2.87 μm corresponding to ^5^I_6_ → ^5^I_7_ transition. Unfortunately, due to its energy level scheme and no GSA (Ground State Absorption) transitions that overlap with low-cost laser diodes, Ho^3+^ ions cannot be directly excited using 980 nm wavelength radiation. Therefore, ytterbium ions characterized by large absorption cross-section at 980 nm are commonly used as a sensitizer in excitation energy transfer to Ho^3+^ ions [20,21,22,23].

In this paper, emission properties in the mid-infrared region of Ho^3+^/Yb^3+^ co-doped heavy metal oxide glasses based on bismuth-germanate oxides and optical fibre have been investigated and compared. The optimal concentration of co-dopants has been estimated based on the luminescence intensity at 2.87 µm (Ho^3+^:^5^I_6_ → ^5^I_7_ radiative transition). The energy transfer (ET) mechanism between lanthanide ions has been discussed in terms of Yb^3+^ → Ho^3+^ ET efficiency. Due to the high thermal stability (ΔT = 124 °C), wide transparency window (0.51–5.5 µm) and low maximum phonon energy (hω_max_ = 724 cm^−1^) bismuth-germanate glass has been selected as the core glass. The results determine that HMO glass in optical fibre is a promising material for construction of new lasers and fibre amplifiers operating in the mid-infrared range.

## 2. Experiment

Series of glasses with the composition of mol% (60 − x − y) (Bi_2_O_3_-GeO_2_)-40 (Na_2_O-Ga_2_O_3_)-x Ho_2_O_3_-yYb_2_O_3_ were synthesized with the standard melt-quenching method from spectrally pure materials (99.99%, Sigma-Aldrich, St. Louis, MO, USA). Labels and specified lanthanide co-dopants composition have been included in Table 1.

The homogenized powder was placed into a platinum crucible and melted at 1050 °C for 60 min. Modified synthesis method was conducted in electric furnace maintaining vacuum conditions (45–65 mBar) in order to minimalize OH^−^ ions concentration. The glass melt was poured into a brass mold and then subjected to the annealing process at 400 °C for 12 h. Transparent and homogenous glasses without any visible crystallization effects have been obtained. Finally, samples were polished in order to provide a high optical quality surface for spectroscopic measurements. Differential Scanning Calorimetry (DSC) measurement was done using the SETARAM Labsys thermal analyzer (Setaran Instrumentation, Caluire, France) at the heating rate of 10 °C/min. The Fourier Transform Infrared Spectroscopy spectrum was recorded with a Bruker Company (Billerica, MA, USA) Vertex 70v spectrometer. Spectra were collected in 3000–450 cm^−1^ infrared region after 128 scans at 4 cm^−1^ resolution. Emission (using high power semiconductor laser diode λ_exc_ = 980 nm as a pump source) and absorption spectra were collected by Stellarnet GreenWave Spectrometer (Stellarnet Inc., Tampa, FL, USA) (0.44–1.1) μm as well as Acton Spectra Pro 2300i monochromator (Princeton Instruments, Trenton, NJ, USA) with PbS and PbSe detectors (1.1–2.3 and 2.75–3.05, respectively) μm with 0.5 nm spectral resolution. For luminescence decay measurements a PTI QuantaMaster QM40 system (Horiba, Kyoto, Japan) coupled with a tuneable pulsed optical parametric oscillator (OPO) was used, it was pumped by the third harmonic of an Nd:YAG laser (OpotekOpolette 355 LD, Carlsbad, CA, USA). The Nd:YAG laser system was equipped with a multi-mode UV−VIS PMT (R928), double 200 mm monochromator and Hamamatsu H10330B−75 detectors (Hamamatsu Photonics K.K., Iwata-City, Japan). Emission decay curves have been recorded with an accuracy of ±1 μs by a PTI ASOC−10 (USB−2500) oscilloscope.

## 3. Results and Discussion

### 3.1. Thermal and Structural Analysis

**Figure 1:**
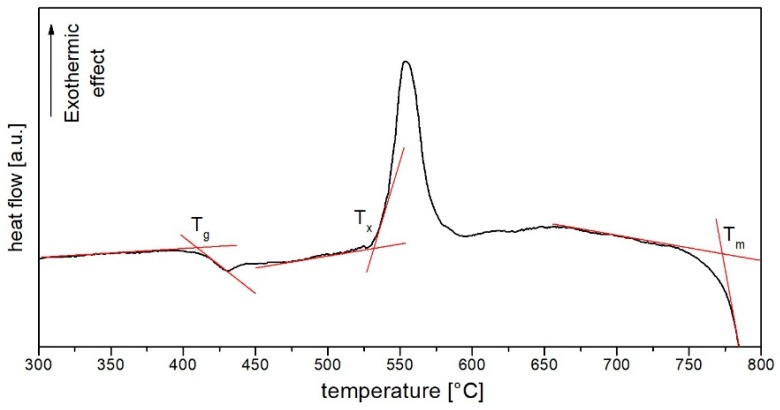
The DSC curve of the produced HMO un-doped glass.

Differential Scanning Calorimetry experimental results of produced host glass have been presented in Figure 1. First endothermic and exothermic peaks, determining the transition temperature T_g_ and crystallization temperature T_x_, have been estimated at 408 °C and 532 °C, respectively, and are comparable to other bismuth (T_g_ = 418 °C; T_x_ = 520 °C) and bismuth-germanate (T_g_ = 442 °C; T_x_ = 550 °C) glasses [24,25]. The high value of thermal stability parameter (ΔT = T_x_ − T_g_ = 124 °C) confirms that investigated glass could be used in the optical fibre drawing process. Low melting temperature T_m_ = 773 °C is an additional advantage, which further simplifies the production process and forming the material into new photonic structures.

**Figure 2:**
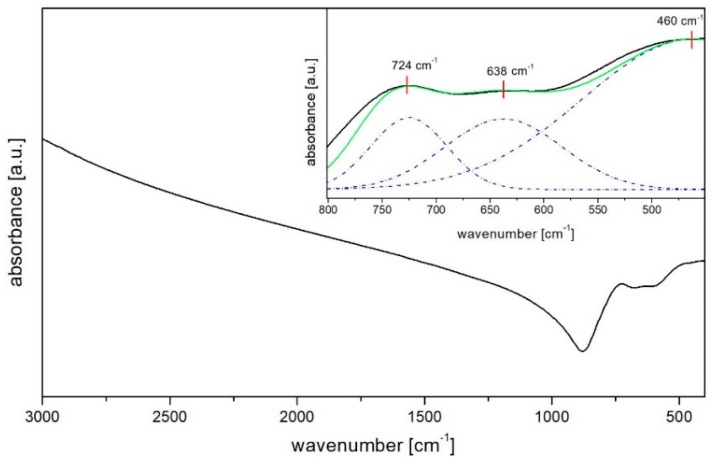
FTIR spectrum of host bismuth-germanate glass and (inset) decomposed 450–800 cm^−1^ region.

Besides high thermal stability essential in optical fibre technology, the composition of bismuth-germanate glass has been developed in terms of low phonon energy. Fourier Transform Infrared Spectroscopy measurements were used to evaluate the structural information of developed amorphous material (Figure 2). In the Fourier-Transform Infrared Spectroscopy spectrum observed in 450–800 cm^−1^ region, which has been presented in the Figure 2 inset, three strong and broad bands have been identified after the decomposition process. The peak at around 460 cm^−1^ originates from bending Bi–O bonds vibrations in BiO_6_ hexahedral units [26,27], while the peak at the 638 cm^−1^ is attributed to stretching vibrations of Bi–O bonds (non-bridged oxygen) in BiO_6_ pyramidal units [28]. The third peak at the wavenumber of 724 cm^−1^ is a result of the motion of Ge–O two non-bridged oxygen atoms in GeO_4_ tetrahedral units [29,30], thereby this absorption band indicates the maximum phonon energy (MPE) of produced bismuth-germanate host glass. The value of MPE is smaller in comparison to silicate (963 cm^−1^) [31], germanate (1002 cm^−1^) [32], phosphate (1345 cm^−1^) [33], borate (1602 cm^−1^) [34], and comparable to other bismuth glasses (926–1183 cm^−1^) [35,36].

### 3.2. Transmission, Absorbance Spectra, and Optical Bandgap Analysis

**Figure 3:**
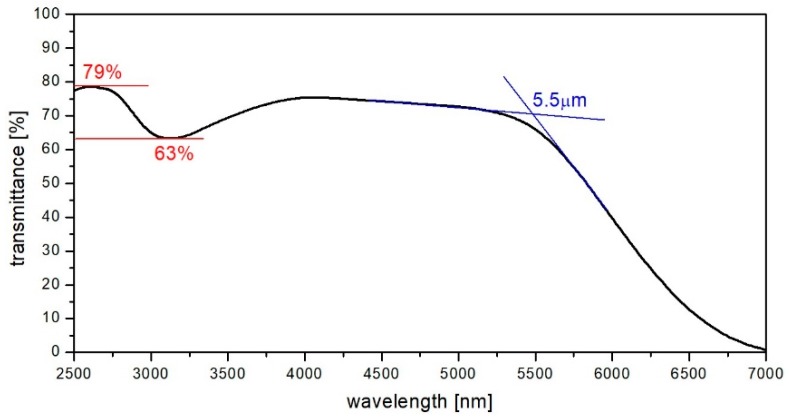
Transmittance spectrum of synthesized host glass in mid-infrared range.

Synthesized host glass has been investigated in respect of mid-infrared transmission parameters. The mid-infrared transmission spectra shown in Figure 3 provide information about hydroxide group content within the structure of produced bismuth-germanate glass, which has been synthesized in a low vacuum environment (45–65 mBar) during the whole thermal process.

The hydroxide ions concentration and the absorption coefficient at the wavelength of 3.1 µm and can be estimated in accordance with the following equations [10]:

(1) $$\alpha_{OH^{-}}=\frac{1000}{l}log\frac{T}{T_{b}}=47\;ppm,$$

(2) $$\alpha_{OH^{-}}=\frac{1}{l}ln\frac{T}{T_{b}}=0.11\;cm^{-1},$$

where *l*—sample thickness (2.0 cm), *T*—the value of the transmittance in the absorption peak (63%), and *T_b_*—the value of the transmittance before absorption peak (79%). The estimated value of the hydroxide groups content and absorption coefficient has been calculated to be 47 ppm and 0.11 cm^−1^, respectively, which are lower than in any known oxide glasses [30,37,38,39]. Hence, the low value of OH^−^ concentration, which acts in a glass matrix as fluorescence-quenching centers, indicates better mid-IR parameters and make the synthesized glass a promising material for mid-infrared applications.

**Figure 4:**
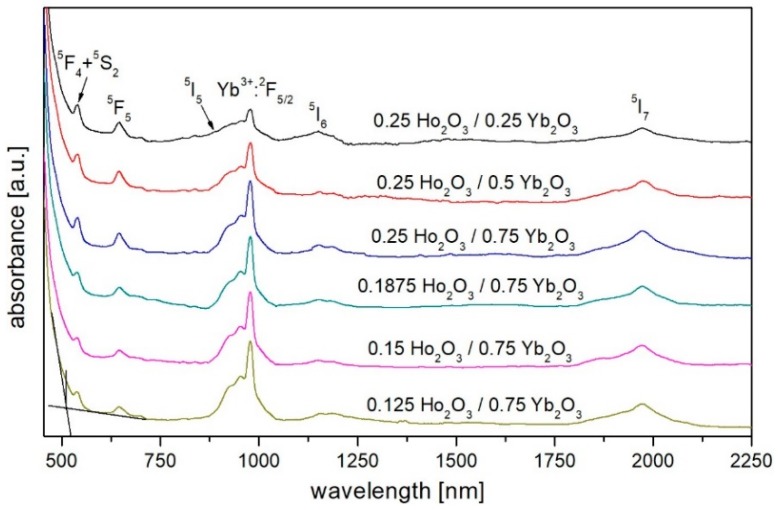
The absorbance spectrum of bismuth-germanate glasses co-doped with holmium and ytterbium ions.

Figure 4 shows the optical absorption spectra of developed glasses with different rare earth ions concentrations. The main absorption bands centered at 540, 645, 890, 1150, and 1972 nm associated to the ground state absorption transitions in holmium ions from ^5^I_8_ level to the higher energy levels (^5^F_4_ + ^5^S_2_), ^5^F_5_, ^5^I_5_, ^5^I_6_, and ^5^I_7_, respectively. The absorption band at 979 nm (Yb^3+^: ^2^F_7/2_ → ^2^F_5/2_ transition) was used to pump ytterbium ions (λ_exc_ = 980 nm) which act as a donor in Yb^3+^/Ho^3+^ system.

Regarding transmittance and absorbance spectra, the transmission window of produced glasses starts from 0.51 µm and reaches approximately 5.5 µm. Due to the large molar content of Bi_2_O_3_ within the glass matrix, UV absorbance band is shifted to the longer wavelengths in comparison to other oxide glasses [31,40,41], due to the reduced interatomic bond strength between the oxygen and metal [42].

**Figure 5:**
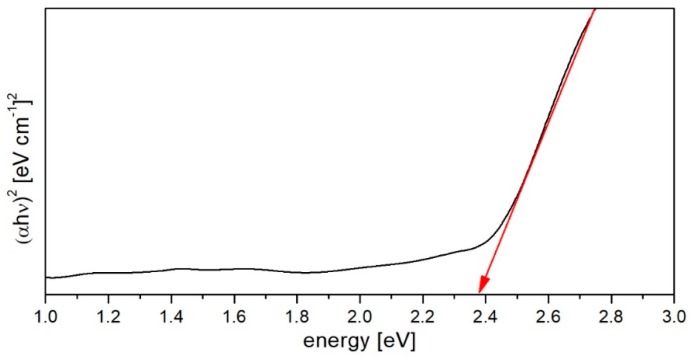
The optical bandgap energy evaluation of (αhν)^2^ vs photon energy plot.

The transmittance spectra absorption edges are used to investigate the electronic band structure and the optical transition of the non-crystalline and crystalline materials. The optical bandgap of amorphous material can be obtained by plotting (αhν)^1/γ^ against incident photon energy hν according to the Tauc’s law [42] (Figure 5):

(3) $${\left(\alpha h\upsilon \right)}^{1/\gamma }=A\left(h\upsilon -E_{g}\right),$$

where *α* is the linear absorption coefficient, A determines band edge parameter, *E_g_* gives optical band energy, *γ* is index number denotes the type of electronic transition which cause the absorption. In the analysed case for allowed indirect transitions (*γ* = 1/2) the value of optical bandgap *E_g_*, obtained from the *(αhν)^1/γ^* vs *hν* variation and the linear extrapolation of the plotted curve, are found to be 2.38 eV, which is typical for the heavy metal oxide glasses [43,44].

### 3.3. Luminescent Properties

**Figure 6:**
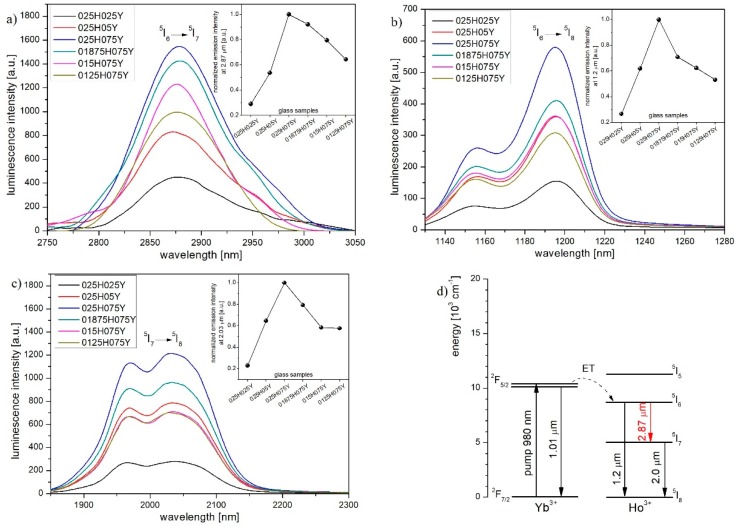
Luminescence spectra of fabricated glasses and normalized emission intensity (insets) in (**a**) 2.87 µm, (**b**) 1.2 µm, (**c**) 2.03 µm wavelength bands and (**d**) simplified Ho^3+^/Yb^3+^ energy level diagram with energy transfer mechanisms.

As a result of pumping at 980 nm (P_opt_ = 1 W), four luminescence bands in the mid-infrared region were observed. Figure 6a presents the emission spectra of lanthanide-doped glasses with a luminescence band centered at 2.87 µm, which could be attributed to the ^5^I_6_ → ^5^I_7_ radiative transition in holmium ions [45]. In samples with a constant concentration of holmium ions (0.25 mol%) and increased content of ytterbium ions (samples 025H025Y → 025H075Y), we observed a significant enhancement of emission intensity to a maximum value. This phenomenon is related to a smaller distance between donor and acceptor ions, which leads to efficient energy transfer from the excited ^2^F_5/2_ energy level of ytterbium. Simultaneously, increased concentration of Yb^3+^ ions results in higher absorption coefficient at the 980 nm wavelength (Yb^3+^: ^2^F_7/2_ → ^2^F_5/2_). Reducing Ho^3+^ ions content and constant Yb^3+^ ions concentration of 0.75 mol% (samples 025H075Y → 0125H075Y) caused mid-infrared luminescence intensity decrease due to the lower concentration of acceptor ions and lower energy transfer efficiency. Moreover, lower holmium concentration in the glass matrix results in the higher distance between acceptor and donor ions. Analogous changes in the emission intensity at 1.2 µm and 2.03 µm bands were observed (Figure 6b,c). It is worth to note that luminescence in 2.87 µm and 1.2 µm infrared bands results from the depopulation of the same ^5^I_6_ excited level via radiative transitions (2.87 µm: ^5^I_6_ → ^5^I_7_ and 1.2 µm: ^5^I_6_ → ^5^I_8_), therefore the changes of maximum emission intensity are similar in the investigated co-doped samples (Figure 6a,b insets). Due to the dynamics of the ^5^I_6_ → ^5^I_7_ transition, preceding transition from the higher excited state ^5^I_6_, which populates the ^5^I_7_ level (Figure 6c inset) luminescence intensity changes in the 2.0 µm band (^5^I_7_ → ^5^I_8_) are compatible.

Obtained results indicate that ET: ^2^F_5/2_ (Yb^3+^) + ^5^I_8_ (Ho^3+^) → ^2^F_7/2_ (Yb^3+^) + ^5^I_6_ (Ho^3+^) energy transfer efficiency (Figure 6d) reaches the highest value in glass co-doped with optimal molar composition of 0.25 Ho_2_O_3_ and 0.75 Yb_2_O_3_ in terms of mid-infrared luminescence. A significant role in the enhancement of mid-IR emission has a distance between lanthanide ions in the glass matrix. Reducing the distance between active centers by increasing the content of lanthanide ions, the probability of the ET: Yb^3+^ → Ho^3+^ and further emission intensity are increased [46].

**Figure 7:**
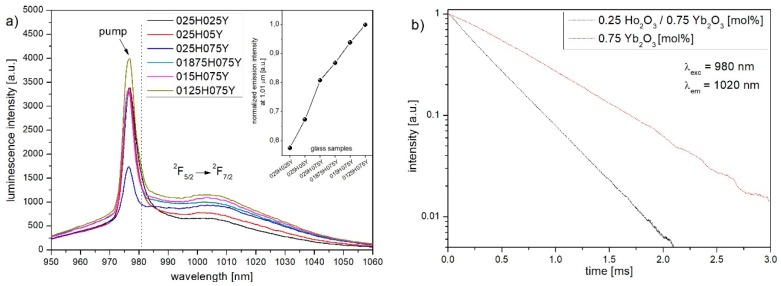
(**a**) Emission spectrum in 1.01 µm band and (**b**) single exponential luminescence decay curves of Yb^3+^:^2^F_5/2_ state in glasses doped with Yb^3+^ and co-doped with Ho^3+^/Yb^3+^ ions.

To further validate the energy transfer process between ytterbium and holmium ions, emission spectra, and fluorescence lifetimes of Yb^3+^: ^2^F_5/2_ state at the wavelength of 1010 nm, which corresponds to ^2^F_5/2_ → ^2^F_7/2_ radiative transition, under 980 nm excitation has been observed and presented in Figure 7. Luminescence intensity at 1.01 µm band increasing from 025H025Y to 0125H075Y which is the result of: 1) higher amount of Yb^3+^ ions in material and higher absorption cross-section at the 980 nm wavelength and 2) lower concentration of Ho^3+^ ions which decreases the donor → acceptor energy transfer efficiency. In Figure 7b, it can be seen that the lifetime in Ho^3+^/Yb^3+^ co-doped sample (τ_HY_) of 266 µs is significantly shorter in comparison to the lifetime of Yb^3+^: ^2^F_5/2_ level in the single-doped glass where τ_Y_ amounts 684 µs. The decrease of the Yb^3+^: ^2^F_5/2_ lifetime indicates the presence of an energy transfer process between holmium and ytterbium ions. Based on the measured lifetime values, ET efficiency has been calculated by the following equation [47]:

(4) $$\eta_{ET}=1-\frac{\tau_{HY}}{\tau_{Y}}=61\%.$$

Estimated energy transfer efficiency equals to 61% which is higher than of other bismuth-germanate (40%) and comparable to germanate glasses (69–71%) [20,48,49]. This indicates that produced Ho^3+^/Yb^3+^ heavy metal oxide glass is a potential material for an active optical fibre core operating in the wavelength range of 2.87 µm.

### 3.4. Active Optical Fibre Co-Doped with Ho^3+^/Yb^3+^ Ions

In accordance with the glass emission measurement results, 025H075Y glass was used as an active core in optical fibre, while the silicate glass (NA = 1.61) was used as a cladding. The refractive index of the active core is equal to 2.19, which establish the numerical aperture NA close to 1. The manufactured optical fibre is characterized by 120 µm core diameter and 250 µm clad diameter. High numerical aperture and large core/clad diameter ratio allow efficient excitation with pumping radiation due to a high absorption coefficient.

**Figure 8:**
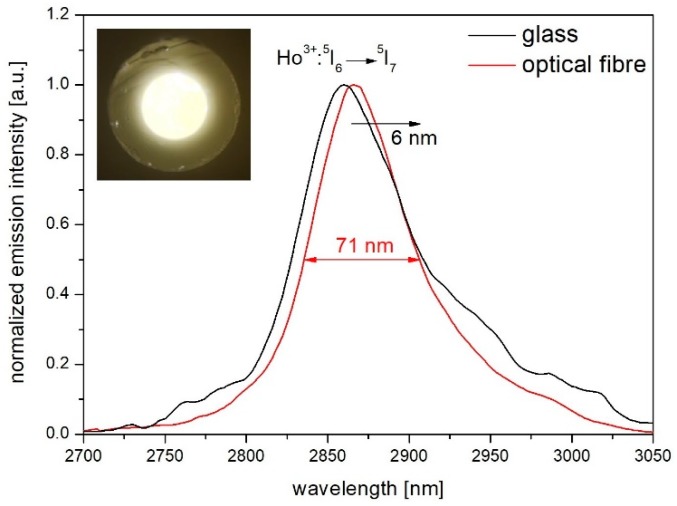
Obtained normalized mid-infrared luminescence spectra and cross section of developed optical fibre (inset).

Figure 8 shows the emission band obtained in the produced optical fibre (*l* = 7 cm) at the wavelength of 2.87 μm under 980 nm pump radiation was presented. As a result of luminescence characterization, the value of Full Width at Half Maximum decreases from 80 nm in the glass to 71 nm in the optical fibre. Narrowing the emission spectrum band, as well as a redshift of the luminescence spectrum, occurs due to the reabsorption of the generated signal which propagates along the fibre core [50].

## 4. Conclusions

In the article, thermal stability, structural, and spectroscopic properties of a low phonon (hω_max_ = 724 cm^−1^) heavy metal oxide glass and optical fibre with core co-doped with Ho^3+^/Yb^3+^ have been investigated. High thermal stability (ΔT = 124 °C) enables forming the fabricated glass into optical fibres, while low hydroxide content in the glassy matrix (47 ppm) provided high transparency in the mid-IR region. Based on the measurements of luminescence parameters the optimal content of co-dopants is 0.25 Ho_2_O_3_/0.75 Yb_2_O_3_ molar percentage in developed heavy metal oxide glass for which high emission intensity in the 2.87 µm band has been observed. To further examine Yb^3+^ → Ho^3+^ energy transfer mechanism decay, lifetimes measurements were carried out demonstrating ET efficiency equal to 61% in the 025H075Y glass sample which has been used as an active core in the optical fibre. Comparison of the luminescence spectra in bulk glass and optical fibre showed a reduction of FWHM from 80 to 71 nm and a redshift of 6 nm towards longer wavelengths in the optical fibre due to the reabsorption of the generated signal phenomenon. The obtained experimental results indicate the possibility of use developed optical fibre with Ho^3+^/Yb^3+^ co-doped core in the mid-infrared applications such as lasers or optical amplifiers.

## Figures and Tables

**Table 1:** The molar percentage of holmium and ytterbium oxides co-dopants.

Glass Sample	Co-Dopants	
	(x) Ho_2_O_3_ [mol%]	(y) Yb_2_O_3_ [mol%]
075Y	0	0.75
025H025Y	0.25	0.25
025H05Y	0.25	0.5
025H075Y	0.25	0.75
01875H075Y	0.1875	0.75
015H075Y	0.15	0.75
0125H075Y	0.125	0.75

## References

[1] Cai M., Wei T., Zhou B., Tian Y., Zhou J., Xu S., Zhang J. (2015). Analysis of energy transfer process based emission spectra of erbium doped germanate glasses for mid-infrared laser materials. J. Alloys Compd..

[2] Guo Y., Tian Y., Zhang L., Hu L., Zhang J. (2013). Erbium doped heavy metal oxide glasses for mid-infrared laser materials. J. Non-Cryst. Solids.

[3] Bai G., Tao L., Li K., Hu L., Tsang Y.H. (2013). Enhanced light emission near 2.7 μm from Er–Nd co-doped germanate glass. Opt. Mater..

[4] Tian Y., Zhang J., Jing X., Zhu Y., Xu S. (2013). Intense mid-infrared emissions and energy transfer dynamics in Ho^3+^/Er^3+^ codoped fluoride glass. J. Lumin..

[5] Mhareb M.H.A., Hashim S., Ghoshal S.K., Alajerami Y.S.M., Saleh M.A., Dawaud R.S., Razak N.A.B., Azizan S.A.B. (2014). Impact of Nd^3+^ ions on physical and optical properties of Lithium Magnesium Borate glass. Opt. Mater..

[6] Boetti N.G., Pugliese D., Ceci-Ginistrelli E., Lousteau J., Janner D., Milanese D. (2017). Highly Doped Phosphate Glass Fibers for Compact Lasers and Amplifiers: A Review. Appl. Sci..

[7] Wang F., Tian Y., Cai M., Jing X., Zhang J., Xu S. (2015). Glass forming ability and enhanced 2.7 μm emission of erbium ions in TeO_2_ doped fluoroaluminate glass. Opt. Mater..

[8] Zhang M., Yang A., Peng Y., Zhang B., Ren H., Guo W., Yang Y., Zhai C., Wang Y., Yang Z. (2015). Dy^3+^-doped Ga–Sb–S chalcogenide glasses for mid-infrared lasers. Mater. Res. Bull..

[9] Tian Y., Wei T., Jing X., Zhang J., Xu S. (2016). Enhanced 2.7- and 2.9-μm emissions in Er^3+^/Ho^3+^ doped fluoride glasses sensitized by Pr^3+^ ions. Mater. Res. Bull..

[10] Tian Y., Xu R., Hu L., Zhang J. (2012). 2.7 μm fluorescence radiative dynamics and energy transfer between Er^3+^ and Tm^3+^ ions in fluoride glass under 800 nm and 980 nm excitation. J. Quant. Spectrosc. Radiat. Transf..

[11] Aly K.A., Abdel Rahim F.M., Dahshan A. (2014). Thermal analysis and physical properties of Bi–Se–Te chalcogenide glasses. J. Alloys Compd..

[12] Li X., Yang B., Zhang J., Hu L., Zhang L. (2014). Energy Transfer between Er^3+^ and Pr^3+^ for 2.7 μm Fiber Laser Material. Fibers.

[13] Pisarski W.A., Grobelny Ł., Pisarska J., Lisiecki R., Ryba-Romanowski W. (2011). Spectroscopic properties of Yb^3+^ and Er^3+^ ions in heavy metal glasses. J. Alloys Compd..

[14] Kityk I.V., Wasylak J., Dorosz D., Kucharski J., Benet S., Kaddouri H. (2001). PbO–Bi_2_O_3_–Ga_2_O_3_–BaO glasses doped by Er^3+^ as novel materials for IR emission. Opt. Laser Technol..

[15] Nian S., Zhang Y., Cao W., Zhenning W., Tang J., Li M., Zhou N., Shu Y. (2018). Optical properties of Er^3+^/Yb^3+^ co-doped bismuth calcium borate glass system for NIR lasers and fiber amplifiers. J. Lumin..

[16] Polosan P. (2017). Crystallisation of bismuth germanate glasses below their glass transition temperature. J. Non-Cryst. Solids.

[17] Pisarski W.A., Pisarska J., Maczka M., Lisiecki R., Grobelny L., Goryczka T., Dominiak-Dzik G., Ryba-Romanowski W. (2011). Rare earth-doped lead borate glasses and transparent glass-ceramics: structure-property relationship. Spectrochim. Acta. Part A Mol. Biomol. Spectrosc..

[18] Żmojda J., Dorosz D., Dorosz J. (2011). 2.1 μm emission of Tm^3+^/Ho^3+^-doped antimony-silicate glasses for active optical fibre. Bull. Pol. Acad. Sci. Tech. Sci..

[19] Ragin T., Zmojda J., Kochanowicz M., Miluski P., Jelen P., Sitarz M., Dorosz D. (2015). 2.7 μm emission in heavy metal oxide glasses doped with erbium ions. Proc. Spie.

[20] Zhao G., Wang S., Fan H., Hu L. (2013). Mid-infrared spectroscopic properties and energy transfer of Er^3+^/Yb^3+^ co-doped bismuth germanate glass. Spectrochim. Acta. Part A Mol. Biomol. Spectrosc..

[21] Żmojda J., Dorosz D., Kochanowicz M., Miluski P., Dorosz J. (2012). Yb^3+^/Ho^3+^-codoped antimony-silicate optical fiber. Proc. Spie.

[22] Zhou B., Tao L., Yat-Yin Chan C., Jin W., Tsang Y.H., Yue-Bun Pun E. (2013). Near- and mid-infrared photoluminescence in Ho^3+^ doped and Ho^3+^–Yb^3+^ codoped low-phonon-energy germanotellurite glasses. J. Lumin..

[23] Bai G., Tao L., Li K., Hu L., Tsang Y.H. (2013). Enhanced ~2μm and upconversion emission from Ho–Yb codoped oxyfluoride glass ceramics. J. Non-Cryst. Solids.

[24] Guo W., Fu L., Lin T., He P., Wang C., Wang T., Liu H. (2019). New design of sapphire joints brazed with bismuth-borate glass. Ceram. Int..

[25] Polosan S., Negrea R., Ciobotaru I.C., Schinteie G., Kuncser V. (2015). Ferromagnetic behaviour of bismuth germanate oxide glass-ceramic materials. J. Alloys Compd..

[26] Pascuta P., Pop L., Rada S., Bosca M., Culea E. (2008). The local structure of bismuth germanate glasses and glass ceramics doped with europium ions evidenced by FT-IR spectroscopy. Vib. Spectrosc..

[27] Dimitrov V., Dimitriev Y., Montenero A. (1994). IR spectra and structure of V_2_O_5_-GeO_2_-Bi_2_O_3_ glasses. J. Non-Cryst. Solids.

[28] Ardelean I., Cora S., Ciceo Lucacel R., Hulpus O. (2005). EPR and FT-IR spectroscopic studies of B_2_O_3_Bi_2_O_3_MnO glasses. Solid State Sci..

[29] Yu Q., Chen F., Xu T., Dai S., Zhang Q. (2013). Glass formation and Raman scattering studies of bismuthate glasses within Bi_2_O_3_–GeO_2_–BaO pseudo-ternary system. J. Non-Cryst. Solids.

[30] Zhao G., Jin W., Fang Y., Gong T., Guo J., Dawai S., Liao M., Hu L. (2016). Broadband mid-infrared emission around 2.9 μm in Dy^3+^ doped bismuth germanate glass. Mater. Res. Bull..

[31] Wang N., Cao R., Cai M., Shen L., Tian Y., Huang F., Xu S., Zhang J. (2017). Ho^3+^/Tm^3+^ codoped lead silicate glass for 2 μm laser materials. Opt. Laser Technol..

[32] Kochanowicz M., Żmojda J., Miluski P., Ragin T., Pisarski W.A., Pisarska J., Jadach R., Sitarz M., Dorosz D. (2017). Structural and luminescent properties of germanate glasses and double-clad optical fiber co-doped with Yb^3+^/Ho^3+^. J. Alloys Compd..

[33] Yaacob S.N.S., Sahar M.R., Sazali E.S., Mahraz Z.A., Sulhadi K. (2018). Comprehensive study on compositional modification of Tb^3+^ doped zinc phosphate glass. Solid State Sci..

[34] Hivrekar M.M., Sable D.B., Solunke M.B., Jadhav K.M. (2018). Different property studies with network improvement of CdO doped alkali borate glass. J. Non-Cryst. Solids.

[35] Zhao G., Tian Y., Wang X., Fan H., Hu L. (2013). Spectroscopic properties of 1.8μm emission in Tm^3+^ doped bismuth silicate glass. J. Lumin..

[36] Krishnan M.L., Neethish M.M., Ravi Kanth Kumar V.V. (2018). Structural and optical studies of rare earth-free bismuth silicate glasses for white light generation. J. Lumin..

[37] Cai M., Zhou B., Tian Y., Zhou J., Xu S., Zhang J. (2016). Broadband mid-infrared 2.8 μm emission in Ho^3+^/Yb^3+^-codoped germanate glasses. J. Lumin..

[38] Wang C., Tian Y., Li H., Liu Q., Huang F., Li B., Zhang J., Xu S. (2017). Mid-infrared photo-luminescence and energy transfer around 2.8 μm from Dy^3+^/Tm^3+^ co-doped tellurite glass. Infrared Phys. Technol..

[39] Cao R., Lu Y., Tian Y., Huang F., Guo Y., Xu S., Zhang J. (2017). Mid-infrared luminescence and energy transfer of Tm^3+^ in silicate glasses by codoping with Yb^3+^ ions. Opt. Laser Technol..

[40] Sajna M.S., Perumbilavil S., Prakashan V.P., Sanu M.S., Joseph C., Biju P.R., Unnikrishnan N.V. (2018). Enhanced resonant nonlinear absorption and optical limiting in Er^3+^ ions doped multicomponent tellurite glasses. Mater. Res. Bull..

[41] Chelcea R., Rada S., Culea E., Coroiu I. (2016). The change of the local environment of MnO incorporated in the lead-germanate glassy network. J. Non-Cryst. Solids.

[42] Biswas K., Sontakke A.D., Sen R., Annapurna K. (2013). Enhanced 2 μm broad-band emission and NIR to visible frequency up-conversion from Ho^3+^/Yb^3+^ co-doped Bi_2_O_3_-GeO_2_-ZnO glasses. Spectrochim. Acta. Part A Mol. Biomol. Spectrosc..

[43] Rada M., Rus L., Rada S., Culea E., Rusu T. (2014). The network modifier and former role of the bismuth ions in the bismuth–lead-germanate glasses. Spectrochim. Acta Part A Mol. Biomol. Spectrosc..

[44] Madheshiya A., Gautam C., Upadhyay S. (2018). Preparation, optical and electrical properties of bismuth substituted lead titanate borosilicate glass and glass ceramics. J. Non-Cryst. Solids.

[45] He J., Zhou Z., Zhan H., Zhang A., Lin A. (2014). 2.85µm fluorescence of Ho-doped water-free fluorotellurite glasses. J. Lumin..

[46] Zhou B., Wei T., Cai M., Tian Y., Zhou J., Deng D., Xu S., Zhang J. (2014). Analysis on energy transfer process of Ho^3+^ doped fluoroaluminate glass sensitized by Yb^3+^ for mid-infrared 2.85 μm emission. J. Quant. Spectrosc. Radiat. Transf..

[47] Zhang P., Hang Y., Zhang L. (2012). Deactivation effects of the lowest excited state of Ho^3+^ at 2.9 µm emission introduced by Pr^3+^ ions in LiLuF_4_ crystal. Opt. Lett..

[48] Tao L., Zhou B., Bai G., Wang Y., Hao J., Tsang Y.H. (2013). Broadband conversion of ultraviolet to visible and near-infrared emission in Gd3+/Yb3+ codoped germanate glass. J. Non-Cryst. Solids.

[49] Kochanowicz M., Zmojda J., Miluski P., Baranowska A., Ragin T., Dorosz J., Kuwik M., Pisarski W.A., Pisarska J., Leśniak M. (2019). 2 µm emission in gallo-germanate glasses and glass fibers co-doped with Yb^3+^/Ho^3+^ and Yb^3+^/Tm^3+^/Ho^3+^. J. Lumin..

[50] Kochanowicz M., Zmojda J., Dorosz D. (2014). Fluorosilicate and fluorophosphate superfluorescent multicore optical fibers co-doped with Nd^3+^/Yb^3+^. Opt. Fiber Technol..

